# Hybrid Tumor of the Parotid Gland: A Case Report and Review of the Literature

**DOI:** 10.1155/2015/192453

**Published:** 2015-02-09

**Authors:** Alain Sabri, Ibrahim Bawab, Ibrahim Khalifeh, Elie Alam

**Affiliations:** ^1^Chair, Otolaryngology Head and Neck Surgery Cleveland Clinic Abu Dhabi, P.O. Box 112412, Abu Dhabi, UAE; ^2^Department of Otolaryngology Head and Neck Surgery, American University of Beirut Medical Center, P.O. Box 11-0236, Riad El Solh, Beirut 1107-2020, Lebanon; ^3^Department of Pathology and Laboratory Medicine, American University of Beirut Medical Center, P.O. Box 11-0236, Riad El Solh, Beirut 1107-2020, Lebanon

## Abstract

The parotid gland is the most common location of benign neoplasms affecting major salivary glands. Hybrid tumors are very rare tumor entities which are composed of two different tumor types, each of which conforms to an exactly defined tumor category. The tumor entities of a hybrid tumor are not separated but have an identical origin within the same topographical area. This report describes a 51-year-old male with three neoplasms occurring within a single parotid gland tumor. The clinical, radiological, and histologic features are described in addition to a review of the literature.

## 1. Introduction

The parotid gland is the most usual location of benign neoplasms affecting major salivary glands and quite often the recurrence of these tumors is noticed, especially in the case of pleomorphic adenoma.

When synchronous tumors of the parotid gland are encountered, the most common histology is that of multiple Warthin's tumors [1]. Multifocal primary tumor (MPT) in the parotid gland is a rare phenomenon [2, 3]; when it occurs, the most common combination is Warthin tumor and a pleomorphic adenoma [4, 5]. Hybrid carcinomas of the salivary gland are a recently defined and rare tumor entity, consisting of two histologically distinct types of carcinoma within the same topographic location [6].

Hybrid tumors must also be distinguished from the multiple occurrences of salivary gland tumors which can develop syn- or metachronously.

To our knowledge, no cases in the literature mentioned the occurrence of a hybrid tumor consisting of three different carcinomas in a single parotid gland.

## 2. Case Report

A 51-year-old male patient, smoker, known to have diabetes insipidus type II, presented with a large deforming right hemifacial mass growing progressively over a period of 10 years, measuring 11 × 14 × 6 cm extending from the level of the right temporalis muscle above the zygomatic arch superiorly, down to the level of the mandible inferiorly, medially extending to the right lateral epicanthal fold, and laterally abutting the right external auditory meatus. The mass was firm, fixed but not tender, rubbery consistent with no overlying skin changes. Weakness was noted over the right eyebrow and right lower lip. No trismus or respiratory distress was present. Oral exam was unremarkable and a fiberoptic flexible laryngoscopy showed no narrowing of the nasopharyngeal, oropharyngeal, or hypopharyngeal walls. Narrowing of the right external auditory canal was evident on otoscopic exam. To note that the patient underwent a previous surgery in an outside hospital in attempt of removing the tumor bulk but the resection was minimal and a small portion of the tumor was removed, no pathological studies were taken at the time. 

### 2.1. At Our Center

**Figure 1:**
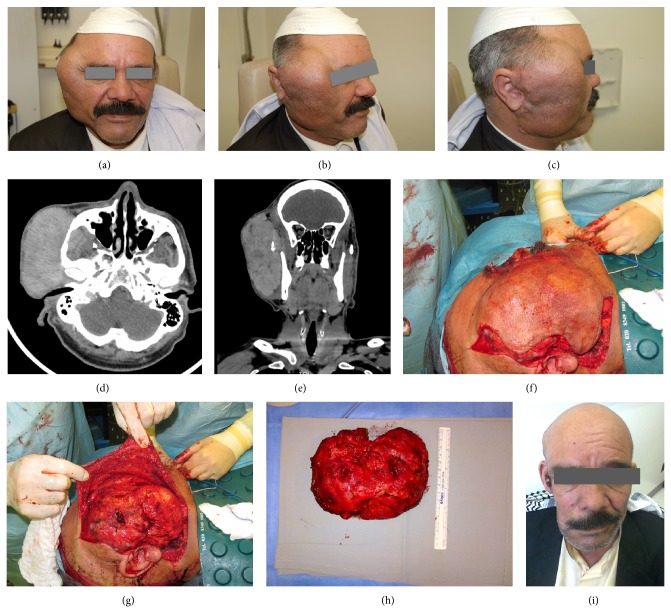
(a) Tumor involving the right cheek area extending from the temporal area to the mandibular area (frontal view). (b) Semilateral view. (c) Lateral view. (d) CT scan: axial cut of the upper neck with IV contrast showing narrowing of the external auditory canal by the tumor. (e) CT scan: coronal cut of the upper neck with IV contrast showing erosion of the right zygomatic arch. (f) Intraoperative image showing an extended modified Blair incision. (g) Intraoperative image showing the parotid mass well circumscribed after elevation of the skin flap. (h) Tumor after “en bloc” excision. (i) Postoperative image of the patient.

CT scan of the brain and neck showed 11 × 6.2 × 14.5 cm heterogeneously enhancing right parotid mass with areas of necrosis, involving the deep lobe of the parotid gland. It is invading the lateral aspect of the masticator space, right temporomandibular joint, and external auditory canal causing narrowing of the latter and extending superiorly into the temporalis muscle and involving the masseter muscle (Figure 1). The parapharyngeal space appears normal. There is erosion of the right zygomatic arch and a few subcentimetric lymph nodes surrounding the parotid mass posteriorly.

A fine needle aspirate was done showing a picture suggestive of pleomorphic adenoma.

The decision was made to surgically excise the mass.

An extended right radical parotidectomy with excision of a portion of temporalis muscle, part of the masseter muscle, and zygomatic arch was made. Intraoperatively, the facial nerve was disappearing into tumor and was completely encased and invaded by tumor; a frozen section biopsy taken intraoperatively demonstrated high suspicion of malignancy, so the decision was made to sacrifice the facial nerve.

### 2.2. Postoperatively

Patient was scheduled for postoperative radiation.

### 2.3. Pathology

**Figure 2:**
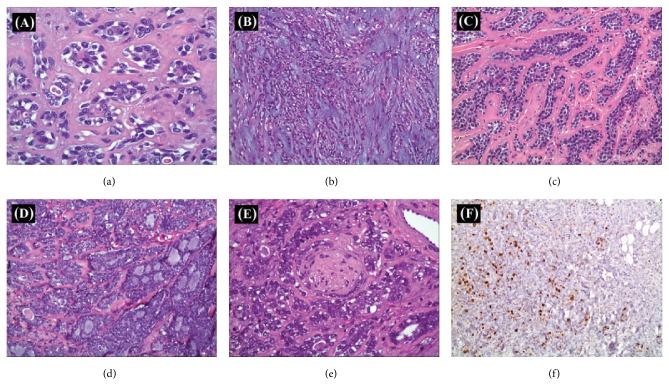
(a) Epithelial-myoepithelial carcinoma. The ductal structures are composed of outer layer of polygonal, clear myoepithelial cells with variable sized and shaped nuclei. The central epithelial cells are smaller and have eosinophilic cytoplasm, round to oval nuclei, and a high nuclear to cytoplasmic ratio. (b) Focally, the epithelial-myoepithelial carcinoma component exhibits myxochondroid rich background with anastomosing stands of epithelial cells reminiscent of pleomorphic adenoma morphologically. (c) Basal cell adenocarcinoma: prominent eosinophilic hyaline material is seen around nodules of tumor composed of small cells with more chromatic nuclei around larger cells with paler nuclei. (d) Adenoid cystic carcinoma: tubular and cribriform and solid tumor nests. Basal lamina-containing cyst-like spaces are noted. The tumor cells show high grade cytology with markedly increased nuclear to cytoplasmic ratio. (e) Nests of tumor invading the peripheral portion of the nerve bundle in the areas dominated by adenoid cystic carcinoma. (f) The proliferative index by Ki-67 stain labeling showed significantly higher labeling index in the adenoid cystic component (lower left corner) in comparison to the epithelial-myoepithelial component (upper right corner).

Mass is composed of multiple components, with epithelial-myoepithelial carcinoma being dominant (80% of the tumor mass) with a minor component of solid variant of adenoid cystic carcinoma and basal cell adenocarcinoma (Figure 2). The diagnosis of a hybrid carcinoma was made, in the presence of perineural invasion and absence of lymphovascular invasion. The surgical margins were negative for malignancy.

## 3. Discussion

Very few articles have described the presence of synchronous parotid tumor. Unilateral synchronous neoplasms of the parotid gland are rare. The incidence ranges from 0.2% to 0.7% of parotid gland tumors [1–8].

The combination most commonly seen is pleomorphic adenoma and a Warthin's tumor [9]. Hybrid tumors are very rare tumor entities which are composed of two or more different tumor types, each of which conforms to an exactly defined tumor category. The tumor entities of a hybrid tumor are not separated but have an identical origin within the same topographical area [10].

In most cases adenoid cystic carcinoma has been the predominant component in hybrid carcinomas [11]. In contrast, biphasically differentiated tumors are a mixture of two cellular patterns with a corresponding term in the tumor classification. Examples of a biphasic differentiation are basaloid-squamous carcinoma, adenosquamous carcinoma or sarcomatoid carcinoma, and epithelial-myoepithelial carcinoma, mucoepidermoid carcinoma, or adenoid cystic carcinoma. Hybrid tumors must also be distinguished from the multiple occurrences of salivary gland tumors which can develop syn- or metachronously [10].

In 1996, Seifert and Donath reported 5 cases of hybrid tumors between 1965 and 1994 in the tissue samples of more than 6600 salivary gland tumors covered by the Salivary Gland Register (Institute of Pathology, University of Hamburg, Germany). This means less than 0.1% of all registered tumours [10].

Moreover, in 2002, Nagao et al. described nine cases of hybrid carcinomas. The combinations of carcinoma components in their report were as follows: epithelial-myoepithelial carcinoma and basal cell adenocarcinoma in two cases, epithelial-myoepithelial carcinoma and squamous cell carcinoma in one case, salivary duct carcinoma and adenoid cystic carcinoma in two cases, myoepithelial carcinoma and salivary duct carcinoma in one case, acinic cell carcinoma and salivary duct carcinoma in one, and squamous cell carcinoma and salivary duct carcinoma in two cases. The prevalence of hybrid carcinomas was 0.4% among the parotid gland tumors in their series [6].

Additionally, in 2003, Ruíz-Godoy et al. reported two patients with hybrid tumours in minor salivary glands of the palate. The first case involved adenoid cystic carcinoma and mucoepidermoid carcinoma, and the second case exhibited adenoid cystic carcinoma and epithelial-myoepithelial carcinoma [11].

In 2010, Kainuma et al. described a case of a 74-year-old male with a hybrid carcinoma composed of epithelial-myoepithelial and salivary duct carcinomas of the right parotid gland [12].

Basal cell adenocarcinoma is a rare entity that was first defined as a malignant salivary gland tumor in 1991; although histomorphologic features of the tumors are similar to basal cell adenomas, proof of an infiltrative and destructive growth is essential for diagnosis. Due to their biologic behavior and prognosis, basal cell adenocarcinomas should be classified as low-grade carcinomas [13].

Epithelial-myoepithelial carcinoma (EMC) is an uncommon epithelial neoplasm, comprising approximately 1% of all salivary gland tumors. The tumor is mainly composed of variable portions of ductal and clear staining myoepithelial cells. EMC is predominantly a tumor of the major salivary glands, especially the parotid gland, but they may also arise in minor salivary glands [14–16].

The aspirates of epithelial-myoepithelial carcinomas have been frequently misread as pleomorphic adenoma [17]. A dual cell population representing duct epithelial and myoepithelial cells with stromal substance is a feature common to both epithelial-myoepithelial carcinoma and pleomorphic adenoma. Although the presence of the double-layered tubules consisting of duct epithelial cells surrounded by myoepithelial cells is diagnostic of epithelial-myoepithelial carcinoma, this pattern is not consistently observed in aspirates of epithelial-myoepithelial carcinomas.

Because of the tendency of local recurrence and the low metastatic potential, the tumor is now recognized to be a low-grade malignant tumor in the WHO salivary gland classification.

The importance of this case lies in the rarity to see simultaneous different cancers within the parotid gland and to educate physicians that treatment for such cases is towards the tumor with the most aggressive type. Moreover, this case introduces epithelial-myoepithelial carcinoma and stresses that high clinical suspicion should be present for such an entity during patient history and disease progression since this type can often be mistaken for pleomorphic adenoma by FNA.
